# Management of Acute Myeloid Leukemia: A Review

**DOI:** 10.3390/cancers18040659

**Published:** 2026-02-18

**Authors:** Chetan Jeurkar, Lana King, David Baek, Lindsay Wilde, Gina Keiffer, Margaret Kasner

**Affiliations:** 1 Sidney Kimmel Comprehensive Cancer Center, Department of Medical Oncology, Sidney Kimmel Medical College, Thomas Jefferson University, Philadelphia, PA 19107, USAmargaret.kasner@jefferson.edu (M.K.); 2 Sidney Kimmel Medical College, Thomas Jefferson University, Philadelphia, PA 19107, USAdavid.baek@students.jefferson.edu (D.B.)

**Keywords:** acute myeloid leukemia, menin inhibitors, *FLT3*, *FLT3* inhibitors, *TP53*

## Abstract

Background/Objectives: Acute myeloid leukemia (AML) is a heterogeneous hematologic malignancy with historically poor outcomes, particularly among older adults and patients harboring high-risk molecular features. Advances in genomic profiling have enabled the development of targeted therapies, reshaping treatment algorithms beyond conventional cytarabine-anthracycline induction and hypomethylating agent-based regimens. This review summarizes current evidence and emerging therapeutic strategies across four evolving areas: menin inhibition, *FLT3* inhibition, *IDH* inhibition and treatment approaches for *TP53*-mutated AML. Methods: We reviewed published clinical trials, preclinical studies, and ongoing clinical trials evaluating targeted therapies in AML. Emphasis was placed on agents with regulatory approval or substantial clinical development, including menin inhibitors, *FLT3* inhibitors, *IDH* inhibitors and novel therapies directed at *TP53*-mutated disease. Mechanistic data, response rates, survival outcomes, and resistance patterns were analyzed to provide an updated overview of therapeutic progress. Results: Menin inhibitors have demonstrated significant activity in *NPM1*-mutated and *KMT2A*-rearranged AML, with agents such as revumenib and ziftomenib producing meaningful remission rates and ongoing studies exploring combination strategies to mitigate resistance. *FLT3* inhibitors, including midostaurin, gilteritinib, and quizartinib, have improved survival in *FLT3*-mutated AML, while emerging evidence supports potential benefit in selected *FLT3*–wild-type disease based on *FLT3*-like gene expression signatures. *IDH* inhibitors, namely ivosidenib and enasidenib, have provided increased efficacy in AML patients carrying these mutations. Questions still remain regarding their efficacy in contrast to venetoclax which has been shown to be particularly effective against this population. In contrast, *TP53*-mutated AML remains a therapeutic challenge: although hypomethylating-agent/venetoclax-based regimens yield improved initial responses, remissions are generally short-lived and overall survival remains poor. Early-phase therapies, including p53 reactivators and multi-kinase inhibitors, show preclinical promise but lack definitive clinical efficacy to date. Conclusions: Targeted therapies have improved outcomes in molecularly defined subsets of AML, with menin, *IDH* and *FLT3* inhibitors representing major advances. However, *TP53*-mutated AML continues to carry a dismal prognosis, underscoring the need for more effective therapeutic strategies. Continued biomarker-driven research, novel drug combinations, and mechanistic insights will be essential to further refine AML treatment and improve long-term survival across disease subsets.

## Simple Summary

Acute myeloid leukemia is a life-threatening blood cancer with many treatment options, but outcomes vary depending on a person’s age and the genetic changes in their cancer cells. While some new therapies have improved remission rates and survival, certain subtypes of leukemia, for example those with *TP53* mutations, remain difficult to treat. This review explores new drug strategies being studied in clinical trials, including therapies that target specific mutations, such as *NPM1*, *KMT2A*, *FLT3* and *IDH1*/2, as well as early research into overcoming resistance in *TP53*-mutated leukemia. The goal is to highlight how these emerging therapies are reshaping treatment approaches and to identify where research is still urgently needed. By summarizing the latest progress and challenges, this work aims to guide future research and improve outcomes for patients with this complex and deadly disease.

## 1. Introduction

Acute myeloid leukemia (AML) is an aggressive malignancy arising from clonal cancer cells in the bone marrow. AML is a relatively rare malignancy with the rate of new cases in the United States being 4.3 per 100,000 individuals. However, mortality from AML remains high with an estimated 33% 5-year relative survival rate [1]. This has significantly improved over time with the advent of novel therapies, both targeted and non-targeted, allowing for deeper eradication of malignant cells. Options for induction treatment are largely determined by molecular characteristics of the tumor and patient characteristics which define eligibility for intensive chemotherapy. The wide adoption of next-generation sequencing (NGS), polymerase chain reaction (PCR), and fluorescence in situ hybridization (FISH) in addition to traditional karyotyping has greatly deepened our understanding of disease processes and drivers of malignancy. This understanding has allowed researchers to develop targeted and therefore more effective therapies.

The first true induction therapy for AML with reliable success was the “7 + 3” regimen consisting of the nucleoside analog cytarabine along with an anthracycline chemotherapy. Cytarabine was given 7 days continuously, while the anthracycline was given for 3 days in three separate doses. The landmark study showing this regimen’s efficacy was from 1973 and was published by Crowther et al. [2], showing a 50% complete remission (CR) rate. The 7 + 3 strategy is still the backbone used in many regimens, and though many other intensive regimens have been developed since (FLAG-Ida, FLAG-Ida-Ven, CLAG-M, CPX-351), it remains an extremely effective therapy for those who can tolerate the intensity of the side effects and cytopenias. CPX-351 is a liposomal formulation of 7 + 3 indicated for the treatment of treatment-related or myelodysplastic-related AML and is approved for patients age 60–75, making it an option for older patients [3].

The next landmark regimen which has been widely adopted in AML practice is the combination of a hypomethylating agent (either azacitidine or decitabine) and the BCL-2 inhibitor, venetoclax, often called “HMA/Ven.” The VIALE-A study published in the New England Journal of Medicine in 2020 showed a CR rate of 36.7% with the combination of azacitidine and venetoclax and importantly showed increased overall survival from 9.6 months to 14.7 months. This study was designed for patients ineligible for intensive chemotherapy regimens or over the age of 75. This study provided an extremely effective and tolerable regimen to unfit or older patients in need of therapy [4].

Novel therapies for AML often use either 7 + 3, CPX-351 or HMA/Ven as backbone chemotherapy and add a targeted drug or one with a novel mechanism of action to increase efficacy and rates of remission. In this review, we will focus on the following therapeutic categories: menin inhibitors, isocitrate dehydrogenase (*IDH*) inhibitors, FMS-like tyrosine 3 (*FLT3*) inhibitors and the most challenging AML to treat, *TP53*-mutated disease, with a focus on pre-clinical work aimed at improving efficacy in this devastating disease.

## 2. Body

### 2.1. Menin Inhibitors

Mechanism of Leukemogenesis: Menin, encoded by the *MEN1* gene, is a nuclear scaffold protein involved in the regulation of gene expression. Loss of function germline mutations in *MEN1* lead to multiple endocrine neoplasia type 1 syndrome, which is a hereditary cancer predisposition syndrome. Accordingly, there is a defined role for menin in the development of acute leukemia due to the function of the protein regarding epigenetic regulation [5]. Menin acts as an adaptor protein that links lysine methyltransferase 2A (*KMT2A*), also known as the mixed-lineage leukemia (*MLL*) gene, to lens epithelium derived growth factor (*LEDF*) [6]. This complex goes on to activate pro-leukemic genes, including *HOX* genes. *HOX* activation is also important for nucleophosmin 1 (*NPM1*)-mutated AML, where *HOX/MEIS1* gene activation via *NPM1* mutation supports a leukemic state [7]. Both mutations in the *NPM1* gene and various rearrangements with *KMT2A* (*KMT2Ar*) activate the *KMT2A*/Menin complex leading to downstream activation of various pro-leukemic genes [5]. Menin inhibitors have been developed to target the menin-*KMT2A* complex and prevent the transcription of leukemogenic genes [8]. Menin inhibitors are small molecule drugs which block menin’s hydrophobic binding site, inhibiting menin-*KMT2A* complex formation and subsequent oncogenic gene activation [5].

Burden of Disease: Mutations in *NPM1* constitute about 20–30% of AML cases, making it one of the most common genetic alterations. *KMT2Ar* AML, on the other hand, accounts for about 5–10% of all AML cases, but patients with *KMT2Ar* disease experience high rates of relapse and resistance to therapy [9,10]. This disease burden reveals an important role for menin inhibitors to target both *NPM1*-mutated (*NPM1*m) AML and aggressive *KMT2A*r AML.

Currently Approved Treatments: As of now, revumenib and ziftomenib are the two FDA-approved menin inhibitors for the treatment of relapsed/refractory AML. Revumeninb is approved for AML with either an *NPM1* mutation or *KMT2A*r. Ziftomenib is approved for *NPM1*-mutated AML only and not *KMT2A*r AML. Revumenib was tested in the phase I/II, open-label, dose-escalation and expansion study AUGMENT-101. Eligible patients had R/R leukemia with either an *NPM1* or *KMT2A*r rearrangement. The phase II arm of the study showed significant results, with a high overall response rate (63.2%) and 68.2% of patients within the composite complete remission group reaching minimal residual disease (MRD) negativity, an important endpoint in the long-term outcomes of this disease [11]. Ziftomenib was tested in the KOMET-001 trial, finding that 22% of patents reached their primary end point of CR/CRh [12]. Ziftomenib will also be utilized in an upcoming study for patients who are treatment-naïve and cannot receive other standard chemotherapy regimens (NCT06930352).

Trials in Progress: There are currently three other menin inhibitor drugs in clinical trials as monotherapy for AML: BN104, enzomenib (DSP-5336) and bleximenib (JNJ-75276617). A phase I/II trial is currently in progress for BN104 (NCT06052813). BN104 is a highly efficient, non-covalent menin inhibitor with a low risk of QTc prolongation and a wide therapeutic window. Results from the phase I dose escalation trial for enzomenib showed no dose-limiting toxicities, with objective response rates of 59.1% and 53.8%, respectively, for *KMT2A*r and *NPM1*m disease (NCT04988555) [13]. Finally, a bleximenib phase I/II trial is in progress (NCT04811560), with preclinical studies showing significant efficacy in vitro and in vivo [14].

Along with monotherapy treatments, menin inhibitors are being robustly investigated as combination therapy with other inhibitors or standard of care chemotherapy regimens. Combination studies are important since menin inhibitors alone have been shown to confer resistance [15]. Revumenib, for example, has been paired with various drug combinations, such as with 7 + 3 and midostaurin for *NPM1*- and *FLT3*-mutated AML (NCT06313437), with venetoclax for MRD+ AML (NCT06284486), and with gilteritinib for *FLT3* and concurrent KMAT2Ar or *NPM1*m AML (NCT06222580). Additionally, the phase I/II SAVE trial tested the combination of revumenib, venetoclax, and decitabine/cedazuridine in patients with R/R AML and found high rates of remission: the 6-month relapse-free survival rate was 59%, and among patients with CR/CRh, 93% reached MRD negativity [16]. Other studies are assessing the array of investigational drugs with less intensive chemotherapy regimens, such as venetoclax and azacitidine. Additionally, some studies are investigating specific indications for menin inhibitors, such as the use of menin inhibitors after allogenic stem cell transplant (NCT06575296).

Pre-Clinical Development: Development of new menin inhibitors is ongoing, with work in the pre-clinical space. There are several other menin inhibitors that have shown efficacy but have yet to go to trial. For example, MI-3454 is a related analog of ziftomenib, and pre-clinical studies in both mouse and patient-derived xenograph models demonstrated that the drug could induce complete remission or regression of *NPM1*m or *KMT2A*r leukemia [17]. Several other novel menin inhibitors went to phase I trials but did not advance. For example, the inhibitor DS-1594 showed pre-clinical efficacy, but the clinical trial was terminated after phase I (NCT04752163) [18].

Summary: The landscape for menin inhibitors is continually evolving. Revumenib and then zifotmenib proved to be an effective treatment for AML, which paved the way for ongoing development of new monotherapies. There have also been promising results from studies that pair novel menin inhibitors with various drug combinations. From these results and ongoing trials, it is evident that the menin complex is an important therapeutic target in the treatment of *NPM1*m and *KMT2A*r AML.

### 2.2. FLT3 Inhibitors

Mechanism of Leukemogenesis: FMS-like tyrosine kinase 3 (*FLT3*) is a class III tyrosine kinase expressed by hematopoietic cells and is responsible for the regulation of cellular functions such as growth, proliferation, apoptosis, and differentiation. Activation of the *FLT3* protein depends on binding of the *FLT3* ligand to the extracellular domain, which induces a conformational change in the protein. Subsequently, dimerization and autophosphorylation of the protein lead to activation of downstream gene pathways. Relevant downstream pathways include *PI3K/AKT/mTOR*, *RAS/MAPK/ERK*, and *JAK/STATFLT3* [19]. Specifically, activation of the *PI3/AKT* and *RAS/ERK* pathways ultimately leads to increased transcription of genes involved in proliferation and supports an anti-apoptotic state [20].

*FLT3* dimerization is normally suppressed when cell differentiation is complete, and this inactive state is maintained by the juxtamembrane domain (JMD) and tyrosine kinase domain (TKD) [19]. Internal tandem duplications (ITD) within the JM domain have been described in both AML and MDS, where they promote abnormal cell growth [21]. Mutations in the activation loop, which is part of the tyrosine kinase domain, were described in later studies and are also present in some patients with AML [22]. Mutations in either domain lead to constitutive activation of the *FLT3* receptor and correspondent downstream gene activation independent of physiologic dimerization.

*FLT3* inhibitors have been employed in the treatment of AML, and they can be classified by generation and type. First generation inhibitors are more broad, whereas second generation inhibitors are more specific to the *FLT3* receptor, which can decrease off-target drug effects [23]. Type I inhibitors bind to the *FLT3* receptor when it is in the active conformation, and these drugs are effective for both ITD and TKD mutations. Type II inhibitors, however, bind the receptor while it is inactive, and they are only effective for ITD mutations [19].

Burden of Disease: The *FLT3* protein is expressed on the cell surface in 70–100% of AML cases, and it is mutated in approximately 30%, making it the most frequently mutated gene in AML. In cases of *FLT3*-mutated AML, ITD changes are more common, comprising approximately 75–80% of cases, whereas TKD changes account for about 20–25%. *FLT3* mutations have historically been associated with a poor prognosis and very proliferative disease often with central nervous system involvement. An important study from 2001 assessed outcomes for 854 patients with *FLT3*-ITD AML mutations and found an increased risk of relapse, higher death rate, and worse overall survival [24]. *FLT3*-ITD mutations have also been shown to have a worse outcome when present with other mutations, such as *NPM1* and DNMT3A. *FLT3*-TKD changes display a similar pattern, with a worse prognosis when combined with DNMT3A and *IDH2*^R140^ mutations [25]. The prognosis for *FLT3*-TKD mutations alone is less clear. Despite historical data indicating poor prognosis for *FLT3*-mutated disease, the prognosis has greatly improved with the advent of *FLT3* inhibitors. A study from 2025 retrospectively reviewed over 600 patient charts examining outcomes for patients with *FLT3*-mutated AML from 2005 to 2023. They found that patients treated with intensive chemotherapy and a *FLT3* inhibitor had a greater overall survival (35.5 months) compared to patients receiving chemotherapy alone (18.9 months) [26]. This marked improvement indicates a vital role for *FLT3* inhibitors in the treatment of AML.

Currently Approved Treatments: In 2017, midostaurin was the first *FLT3* inhibitor agent FDA approved for use in ITD- or TKD-mutated AML. In the RATIFY phase III clinical trial, midostaurin combined with standard chemotherapy induction and consolidation increased overall survival and event-free survival, with a 22% lower risk of death as compared to the placebo group [27]. In 2018, gilteritinib was FDA approved for use in relapsed/refractory AML with ITD or TKD changes after demonstrating an increased median overall survival from 5.6 months to 9.3 months for patients receiving gilteritinib [28]. In a comparison between gilteritinib and midostaurin, the phase II PrECOG 0905 study found that when combined with intensive chemotherapy, patients treated with gilteritinib had higher composite complete remission (Crc) rates than those treated with midostaurin (85.6% vs. 72.4%) [29]. Longer term data, however, is needed to fully assess survival outcomes when comparing the two drugs. There is also a potential role for gilteritinib in the setting of newly diagnosed patients who are unfit for intensive chemotherapy, with the combination of azacitidine, venetoclax, and gilteritinib leading to a favorable 18-month survival rate of 72% in this patient population [30]. The most recent FDA-approved type II inhibitor, as of 2023, is quizartinib. The QuANTUM-First trial demonstrated improved survival (31.9 months vs. 15.1 months) for patients with ITD-mutated, newly diagnosed AML receiving 7 + 3 induction with quizartinib and has become the standard of care for frontline therapy in this patient population [31].

Trials in Progress: Clinical trials are currently investigating other *FLT3* inhibitors for use in AML patients. BMF-500 is a small-molecule *FLT3* inhibitor that showed impressive pre-clinical efficacy, with greater potency than gilteritinib [32]. The phase I trial for BMF-500 is in the recruiting phase (NCT05918692). Another example includes the inhibitor STI-8591 that will be assessed in a phase I dose escalation and dose expansion study (NCT05947344). In vitro, STI-8591 showed 4–7-fold greater antileukemic activity than both gilteritinib and quizartinib on *FLT3*-mutated cells that have been shown to be drug resistant, indicating a potential advantage of the drug for AML with demonstrated *FLT3* drug resistance [33]. MAX-40279 is an inhibitor of both *FLT3* and fibroblast growth factor receptor that is currently in a phase I trial for patients with AML (NCT03412292). Sorafenib is an older broad kinase inhibitor but continues to be tested after improving event-free and relapse-free survival for patients enrolled in the SORAML trial [34]. This is a non-exhaustive list, as additional drugs are also under investigation.

FLT3 Inhibition in FLT3 Wild-Type AML: Aside from targeting *FLT3*-mutated AML, *FLT3* inhibitors have also shown efficacy in the setting of *FLT3* wild-type (WT) disease. It has been shown that the *FLT3* protein is overexpressed, though not derived from an ITD or TKD mutation, on AML cells even in the case of *FLT3*-WT disease [35]. This indicates a potential role for blocking the receptor when it is overactive. Some *FLT3* inhibitors are also multi-kinase inhibitors, which could contribute to their effectiveness in WT disease. The SORAML trial included patients with *FLT3* WT disease, and although the survival outcomes were better for participants with *FLT3*-ITD changes, there was some efficacy shown in WT disease as well [34]. Midostaurin also showed efficacy in WT disease, though a much greater benefit was seen in ITD-mutated AML [27].

Following the results of the SORAML and RATIFY trial, the phase II QUIWI trial tested the combination of chemotherapy (7 + 3) with quizartinib or placebo in patients with *FLT3*-WT AML and included correlative analyses to understand the molecular mechanisms behind response. Analysis showed an estimated event-free survival of 16.6 months for patients receiving quizartinib, as compared to 10.6 months for patients receiving placebo [36]. To help characterize this response, further analysis found that 49.67% of *FLT3*-WT patients had *FLT3*-like gene expression (based on similar RNA sequencing patterns). These patients showed greater event-free survival, relapse-free survival, and overall survival as compared to the placebo group. As such, much of the benefit from *FLT3* inhibition in WT patients in this setting was due to *FLT3*-like gene expression. In the mutational correlates of the patients with *FLT3*-like signatures, they found that 42.5% of patients carried an *NPM1* mutation, 38.7% with a DNMT3A mutation and 23.7% with both *NPM1* and DNMT3A. Furthermore, those who carried either an *NPM1* or DNMT3A mutation (57.5%) drove improved responses to quizartinib (HR = 0.20, *p* = 0.02). Conversely, non-*FLT3*-like signature patients who also carried an *NPM1* or DNMT3A mutation (21%) did not respond better to quizartinib (HR 1.27, *p* = 0.74) [37].

The QuANTUM-Wild study is a large phase III trial which is currently recruiting that aims to confirm the efficacy of combining quizartinib and chemotherapy in the setting of *FLT3*-WT disease [38]. An upcoming trial will assess the combination of midostaurin with revumenib and 7 + 3 in patients with both *FLT3* and *NPM1* mutations (NCT06313437) given the propensity of *FLT3* ITD mutations to co-occur with *NPM1* mutations. Gilteritinib will be assessed in a similar manner for patients with *FLT3* mutations and *NPM1* mutation or *KMT2A* rearrangement (NCT06222580). Information from these studies will be important for assessing novel uses for *FLT3* inhibitors in the setting of various co-mutational states.

Summary: Although the use of *FLT3* inhibitors has improved outcomes in AML patients, challenges remain regarding drug resistance. Patients can either display primary resistance due to pre-existing factors, or they can acquire resistance due to *FLT3* inhibitor use [39]. Combination drug therapies and the use of novel inhibitors may help combat drug resistance, but more definitive work is needed to assess the best drug combinations and treatment plans. *FLT3* inhibition in *FLT3* wild-type AML is a topic of ongoing research and shows promising outcomes.

### 2.3. IDH Inhibitors

Mechanism of Leukemogenesis: Isocitrate dehydrogenase (*IDH*) is an enzyme that has a critical function during the Kreb’s cycle. Physiologically, the *IDH* enzyme catalyzes the oxidative decarboxylation of isocitrate to form α-ketoglutarate, during which NADPH is produced. There are three distinct isoforms of the enzyme, *IDH1*, *IDH2* and *IDH*3. *IDH2* and *IDH*3 catalyze the intra-mitochondrial conversion of isocitrate to α-ketoglutarate, while *IDH1* catalyzes the same reaction but in the cytoplasm. Ultimately, NADPH generated by this reaction is used to generate fatty acids and cholesterol, drive oxidative metabolism of drugs by the cytochrome P450 system, and help generate nitric oxide and reactive oxygen species by neutrophils, which is a critical step in pathogen destruction [40]. In many different cancers, the *IDH* gene is known to be recurrently mutated, and specifically in AML, *IDH1* and *IDH2* are affected. The mutations that are known to occur in AML are in the R132 locus of *IDH1* and the R140, or less commonly the R172 locus, of *IDH2*. Both mutations cause a loss of function in the normal Kreb’s cycle reaction described above and result in the reduction (rather than oxidation) of α-ketoglutarate to 2-hydroxyglutarate (2-HG). 2-HG functions, as Issa et al. describes, as an “oncometabolite” competitively inhibiting α-ketoglutarate dependent pathways importantly leading to a “hypermethylated” phenotype. The aberrant 2HG production also induces BCL2 survival dependence via inhibition of cytochrome C oxidase [41]. These molecular changes induce leukemia cell proliferation and survival, though the unique and specific mechanism by which *IDH* mutations cause AML to make them vulnerable to various therapeutic agents.

Burden of Disease: *IDH1* and *IDH2* mutations occur in approximately 15–25% of AML cases, predominantly in older adults, and are most frequently associated with intermediate-risk cytogenetics. Co-mutations are common, particularly with *NPM1*, DNMT3A, and *FLT3*-ITD, and significantly influence prognosis and therapeutic responsiveness [42,43]. In general, co-mutation with *NPM1* offers a more favorable prognosis, with one study citing near 100% CR rates with either *IDH1*^mut^/*NPM1*^mut^ or *IDH2*^mut^/*NPM1*^mut^ AML with intensive chemotherapy or regimens containing venetoclax. Median overall survival showed a similar trend with *NPM1* mutations co-existing with *IDH* mutations having the longest survival [43]. The impact of co-mutations and the particular sensitivity to venetoclax related to BCL2 survival dependence makes sequencing therapies for *IDH* mutants an ongoing source of debate.

Currently Approved Treatments: At present, *IDH* mutations do not impact the European Leukemia Network (ELN) 2022 risk stratification of AML [44]. Rather, patients with *IDH* mutations are risk-stratified by their cytogenetics, FISH findings and their NGS profile [44]. The decision to transplant therefore depends not solely on their *IDH* mutational status but rather their cytogenetic risk as a whole. Choosing front-line therapy can be challenging. In the VIALE-A trial, patients with *IDH* mutations had a 66.8% overall survival at 12 months with the addition to venetoclax to azacitidine versus 35.7% in the azacitidine group alone [4]. The approval of two *IDH* inhibitors (*IDH*i), ivosidenib for *IDH1* mutations, and enasidenib for *IDH2* mutations, makes choosing front-line therapy more nuanced as there is strong evidence of their benefit in up-front therapy. In patients eligible for intensive chemotherapy, 7 + 3 induction or FLAG-Ida–venetoclax is commonly used, particularly in patients able to tolerate prolonged cytopenias, given higher response rates observed with venetoclax-containing regimens [45]. In patients who are not candidates for intensive chemotherapy, HMA/Ven for either *IDH1*/2 mutants or HMA/Ivosidenib, based on the AGILE trial, for *IDH1* mutants is the treatment of choice [46]. Sequencing venetoclax and *IDH*i is a topic of ongoing research as the AGILE trial [46], AG221-AML-005 [47] and the VIALE-A [4] trial showed excellent efficacy in *IDH1*- and *IDH2*-mutated AML. Ivosidenib and enasidenib as single agents are also approved in the relapsed/refractory setting. Olutasenib is an *IDH1* inhibitor which is a 2b option in the NCCN guidelines for *IDH1* mutant AML in patients ineligible for ivosidenib due to QTC-prolongation [48].

Trials in Progress: Novel combinations of *IDH* inhibitors with other AML therapies is of particular interest, with triplet regimens being at the forefront of trials. NCT05401907 compares sequencing HMA/Ven then HMA/*IDH*i vs. HMA/*IDH*i then HMA/Ven. Treatment failure at 24 months is the primary endpoint, while OS is an important secondary endpoint [49]. This trial will help answer the up-front efficacy question that remains with HMA/Ven vs. HMA/*IDH*i. The ENAVEN-AML trial is a phase 1b/2 trial which looked at enasidenib and venetoclax for *IDH2*-mutated AML. They found that the overall response rate was 62% with 50% achieving a complete remission [50]. NCT03471260 is a phase 1b/2 trial looking at ivosidenib and venetoclax with or without azacitidine in patients with *IDH1*-mutated hematologic malignancies, including AML. The objective of phase 1b is to determine the recommended phase 2 dose (RP2D), while phase 2 was designed to assess overall response rate. This study is actively recruiting and is expected to finish enrollment in 2027 [51]. NCT04774393 is another trial looking at a triplet and all-oral regimen of decitabine/cedazuridine (ASTX727) and venetoclax in combination with ivosidenib or enasidenib for the treatment of refractory acute myeloid leukemia. This study is expected to be completed in 2027 as well [52]. Collectively, these studies aim to define whether early incorporation of *IDH*i improves depth and durability of response compared with venetoclax-based strategies, a question with direct implications for frontline treatment selection.

Drugs in Development: There are several *IDH*i which are being developed in the pre-clinical space and early clinical space which we will discuss here. HMPL-306 (ranosidenib) is a dual *IDH1*/2 inhibitor which demonstrated robust reduction in the oncometabolite 2-HG in mutant *IDH1* and *IDH2* tumor xenograft models [53]. In the phase 1 trial which followed, HMPL-306 showed an acceptable safety profile [54] and is now in phase 3 development. Another *IDH1* inhibitor BAY1436032 was shown to be effective against *IDH1*-mutant AML in two independent patient-derived xenograft *IDH1*-mutant AML models. Importantly, this showed efficacy with all variants of *IDH* mutants [55]. However, in the phase 1 trial, the low overall response rate and incomplete target inhibition did not support further clinical development [56]. The *IDH1* inhibitor HMS-101 is a unique inhibitor targeting the active conformation of the *IDH1* enzyme and showed reduction of 2HG levels and induction of myeloid differentiation in vivo [57]. HMS-101 has not been tested in humans as of yet. SH1572 is a novel *IDH2* inhibitor which showed strong selective inhibition of mutant *IDH2*, effectively reducing 2-HG levels and thereby promoting myeloid differentiation. This pre-clinical data lead to the approval of SH1573 for clinical trial in China [58].

Summary: *IDH1* and *IDH2* mutations occur in approximately 20% of patients with AML and drive leukemogenesis through aberrant production of the oncometabolite 2-hydroxyglutarate, resulting in epigenetic dysregulation, impaired differentiation, and enhanced leukemia cell survival. These mutations create a targetable vulnerability, with *IDH* inhibitors demonstrating clinical efficacy as monotherapy and in combination regimens. The availability of ivosidenib and enasidenib has expanded treatment options across frontline and relapsed/refractory settings, particularly when combined with hypomethylating agents or venetoclax. Emerging data suggest that co-mutation patterns, especially with *NPM1*, significantly influence response and optimal sequencing of therapy. Ongoing trials evaluating triplet and sequencing strategies will be critical in defining the most effective integration of *IDH* inhibitors into AML treatment paradigms.

### 2.4. TP53-Mutated AML

Mechanism of Leukemogenesis: Physiologically, the *TP53* gene encodes the p53 protein that regulates cell cycle arrest, apoptosis, and DNA repair [59]. In response to cellular stress or DNA damage (e.g., ionizing radiation, chemotherapeutics, or oxidative stress), the p53 protein becomes phosphorylated, leading to its activation and subsequent transcription of CDKN1A that results in cell cycle arrest at G1/S and G2/M checkpoints [60]. Then, p53 induces transcription of pro-apoptotic genes such as BAX, PUMA, and p21 to promote mitochondrial outer membrane permeability [48]. For DNA repair, the p53 protein upregulates genes involved in nucleotide excision repair and base excision repair, stabilizing the genome. However, the *TP53* gene can acquire somatic point mutations, deletions, and/or copy-neutral loss of heterozygosity that structurally impairs the p53 protein’s ability to bind DNA and induce transcription of other proteins involved in cell cycle arrest, apoptosis, and DNA repair [42]. By understanding this physiologic role of p53 in cellular repair, we can infer how *TP53* mutations can generate chemotherapy resistance.

Burden of Disease: The *TP53* tumor suppressor gene is one of the most frequently mutated genes in human cancers [61]. Although p53 mutations are found in only 5–10% of de novo AML cases among younger patients [62], they are more frequently observed in elderly patients and found in up to 30% of treatment-related AML [63,64]. The frequency of *TP53* mutations increases even more—up to 70% to 80%—in complex karyotype and/or with loss of chromosome 17/17p, 5/5q, or 7/7q [65]. The mutation confers a very poor risk and is an independent negative prognostic factor for disease-free survival, relapse risk, and overall survival in AML [66,67].

Mechanism of Chemotherapy Resistance: Chemotherapy agents cause DNA damage through various mechanisms, such as the formation of DNA adducts, intrastrand and interstrand cross-links, DNA-protein cross-links, intercalations, and oxidative stress [68]. When such DNA damage occurs in *TP53*-mutant cells, however, the physiologic cellular responses do not occur, leaving the cell unable to produce CDKN1A and pro-apoptotic proteins that allow cell cycle arrest and death. Subsequently, the cell proliferates without inhibition [69,70,71].

There are many reasons why a mutated p53 protein is a difficult therapeutic target. The protein is a nuclear transcription factor that lacks deep pockets required for high binding affinity [72]. The diversity of p53 mutations has also rendered attempts to restore wild-type p53 function, degrade mutant p53, or produce a universal therapeutic largely ineffective. Additionally, mutant p53 proteins frequently build up to high levels in tumor cells and can form abnormal interactions with various cellular partners, which may unintentionally cause off-target effects and development of treatment resistance [73].

Currently Approved Treatments: Therapy for AML with *TP53* mutations remains an unmet need as the conventional intensive chemotherapy regimens have produced poor responses [74]. Lower intensity drugs—namely hypomethylating agents (HMAs) like azacitidine, or decitabine with or without venetoclax—have been investigated [75,76,77]. However, overall response rates among patients with *TP53*-mutated AML were similarly low regardless of which low-intensity regimen was used [56].

Our institution’s current practice as of publishing this review remains either intensive cytarabine- and anthracycline-based chemotherapy or HMA/venetoclax induction followed by possibly an allogeneic stem cell transplant if in CR. However, there are many nuances in each of these decisions that are very patient-specific given the lack of good long-term outcomes. In either situation, relapse rate remains high even after transplant.

In the past five to ten years, various combination therapies have been evaluated for *TP53*-mutated AML, many using venetoclax as part of the regimen. In the landmark VIALE-A trial, the combination of azacitidine with venetoclax showed significantly higher composite remission rates in *TP53-*mutated AML, 55.3%, compared to azacitidine alone, 0%, (*p* < 0.001); however, it did not ultimately improve overall survival [4]. Despite improved composite remissions with the combination regimen, real-world data indicates that remission in *TP53*-mutated AML is not sustained, making the bridge to eventual allogeneic hematopoietic stem cell transplant (HSCT) very challenging [78]. Even with allogeneic HSCT after achieving complete remission with induction therapy, *TP53*-mutated AML prognosis is very poor, often with overall survival less than one year due to the majority of patients relapsing shortly after transplant [79].

Previous Clinical Trials for TP53m AML: APR-246 (eprenetapopt, a small molecule re-activator of mutant p53 protein), studied in combination with azacitidine, showed complete remission in four out of eleven *TP53*-mutant AML patients in a phase Ib/II trial [80]. Despite this promising result, the combination therapy did not improve overall survival during the phase III trial, pausing its commercial development [81]. Another therapy magrolimab, an anti-CD47 antibody, was studied in combination with azacitidine in a phase Ib trial in AML patients among which 65% had *TP53* mutation [82]. Although the antibody showed a 57% complete remission rate, the subsequent phase III ENHANCE-2 clinical trial studying the effects of magrolimab with azacitidine in *TP53*-mutated AML was stopped due to a lack of improvement in overall survival and increased risk of infection and respiratory failure [83]. Lemzoparlimab is another CD47 antibody which targets a different epitope than magrolimab does. This study was done in China and was stopped abruptly for unknown reasons despite having clinical activity in early phase trials [84].

Drugs in Development: There are various other classes of drugs in development for *TP53* AML. We will focus on the following categories: p53 re-activators, novel immunotherapy approaches, and targeted small molecular inhibitors.

P53 re-activators in general restore the tumor suppression function of the p53 protein. This is accomplished by the restoration of the DNA-binding ability of the protein which is often lost when mutated. PC14586 (rezatapopt), a next-generation p53 reactivator, has demonstrated ability to cause massive cell death when combined with venetoclax specifically in *TP53* with Y220C mutations, a mutation that creates a structural pocket for the small molecule to bind and restores the p53 wild-type conformation [68]. PC14586 is now in early-phase clinical trial [85]. A phase 1 trial combining eprenetapopt, as described above, in combination with azacitdine and venetoclax has completed and showed an acceptable safety profile [86]. In the expansion cohort of this trial, overall response rate was found to be 64% (25/39) with a 38% complete remission rate (15/39). In the combined phase 2/long-term follow-up of eprenetapopt + azacitidine in *TP53*-mutant MDS/AML, a high response rate was noted (71%) but still with a median OS of 10.8 months, reinforcing that response does not necessarily mean long-term survival in this disease [80].

Another strategy targeting *TP53* mutations includes novel immunotherapy approaches, though thus far these have shown to either be ineffective or to have no final results available. *TP53*-mutated AML poses unique challenges for all immunotherapy approaches due to tumor evasion from both the adaptive and innate immune systems. P53-deficient tumors employ multiple strategies to avoid T cell-mediated killing including downregulation of MHC class I and II molecules, loss of TRAIL receptors and upregulation of PD-L1 expression. Additionally, *TP53* mutations hinder the anti-tumor functions of the innate immune system through downregulation of NK-activating ligands and conferring resistance to NK-mediated apoptosis [87]. In AML specifically, *TP53*-deficient AML cells upregulate the mevalonate pathway when under CAR-T cell attack, conferring resistance [88]. Due to these evasion mechanisms, *TP53*-mutated AML is a particularly difficult target for immune therapies, namely chimeric antigen receptor therapy (CAR-T) and bispecific T cell engager therapy (BiTE).

Thus far, no CAR-T trial has focused on *TP53*-mutated AML specifically given its known resistance, though many have included *TP53*-mutated patients as part of their cohort in the relapsed refractory setting. The CertainT-1 trial evaluates CER-1236, an autologous chimeric engulfment receptor T-cell targeting TIM-4 ligand, and includes a dedicated expansion cohort for *TP53*-mutated AML patients [89].

BiTE therapy has also been investigated in *TP53*-mutated AML. Flotezumab, a BiTE targeting CD123 and CD3, achieved 47% complete response rate in relapsed/refractory *TP53*-mutated AML, but median overall survival only reached 10.3 months after complete response for this subgroup [90]. Of note, a new T-cell engager, CLN-049, developed by Cullinan Therapeutics, targets an extracellular domain on *FLT3* and is currently in a Phase 1/2 combination study [91]. However, there is no public data yet that shows CLN-049 improves the response or overall survival of *TP53*-mutated AML patients.

In summary, despite several therapeutic developments showing a higher response rate in *TP53*-mutated AML, overall survival has not much improved [63]. It reveals a largely unmet need to better understand the role of *TP53* mutations in causing treatment resistance and new treatment strategies to improve survival in this subset of the AML patient population.

## 3. Conclusions

The treatment landscape of AML continues to evolve with the development of targeted therapies and novel drug combinations built on established backbones such as 7 + 3, CPX-351, and HMA/venetoclax. Menin inhibitors have shown promising efficacy in *NPM1*-mutated and *KMT2A*-rearranged AML, with revumenib’s and ziftomenib’s approvals marking a significant milestone in the field. Ongoing studies exploring combination strategies aim to mitigate resistance, deepen response and move these drugs to the frontline. Similarly, *FLT3* inhibitors have transformed outcomes in *FLT3*-mutated AML, and emerging data suggest potential benefit in *FLT3*-WT disease, expanding their applicability. *IDH* mutations define a biologically distinct and therapeutically actionable subset of AML, with ongoing studies being conducted to refine optimal sequencing and combination strategies to maximize durable remission.

*TP53*-mutated AML remains a significant therapeutic challenge. While certain regimens have improved initial response rates, durable remissions and overall survival remain poor. Novel agents such as p53 reactivators and multi-kinase inhibitors are in early-phase development and may offer a path forward. Continued translational research and biomarker-driven approaches will be critical in refining treatment strategies and improving outcomes across all AML subtypes.

**Figure 1:**
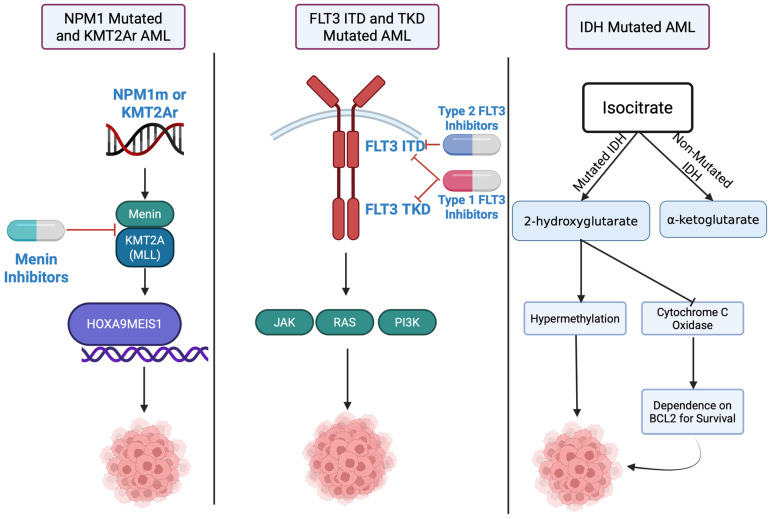
Schematic representation of the major molecular pathways driving leukemogenesis and targeted therapies in acute myeloid leukemia (AML). Black arrows indicate stimulation, red lines with stop bars indicate inhibition. (**Left**) In *NPM1*-mutated and *KMT2A*-rearranged AML, formation of the menin–*KMT2A* transcriptional complex leads to aberrant activation of *HOX* and *MEIS1* genes, sustaining leukemic proliferation and differentiation arrest. Small-molecule menin inhibitors (e.g., revumenib, ziftomenib) disrupt this interaction and suppress oncogenic transcription. (**Middle**) In *FLT3*-mutated AML, internal tandem duplication (ITD) or tyrosine-kinase domain (TKD) mutations result in constitutive activation of *FLT3* and downstream PI3K, RAS, and JAK signaling pathways, driving proliferation and survival. Targeted agents including type 1 inhibitors (midostaurin, gilteritinib) and type 2 inhibitors (quizartinib) block these pathways. (**Right**) In *IDH*-mutated AML, formation of the onco-metabolite 2-hydroxyglutarate (2HG) induces DNA hypermethylation and dependence on BCL2 survival related to cytochrome C oxidase inhibition, leading to AML. Created in https://BioRender.com, accessed on 12 February 2026.

The above presented therapeutic targets with agents and key clinical data are summarized in Table 1. The mechanism of action and leukemogenesis of the three described pathways are shown in Figure 1.

## Figures and Tables

**Table 1:** Summary of currently approved and investigational therapies for *NPM1*-mutated, *KMT2A*r, *FLT3* ITD/TKD-mutated, *IDH1*/2-mutated and *TP53*-mutated AML. Abbreviations: ORR, overall response rate; CR, complete remission; CRh, CR with partial hematologic recovery; MRD, minimal residual disease; OS, overall survival; EFS, event-free survival; ITD, internal tandem duplication; TKD, tyrosine-kinase domain.

Molecular Subtype	Targeted Pathway	Agent	Clinical Setting	Regulatory Status	Key Clinical Data
*NPM1*-mutated AML	Menin–*KMT2A* complex	Revumenib	Relapsed/refractory	FDA-approved	ORR 63.2%; MRD-negative CR/CRh 68.2% (AUGMENT-101)
*NPM1*-mutated AML	Menin–*KMT2A* complex	Ziftomenib	Relapsed/refractory	FDA-approved	CR/CRh 22% (KOMET-001)
*KMT2A*-rearranged AML	Menin–*KMT2A* complex	Revumenib	Relapsed/refractory	FDA-approved	High MRD-negative composite remission rate
*FLT3*-ITD or TKD AML	*FLT3* kinase	Midostaurin	Frontline with 7 + 3	FDA-approved	Improved OS and EFS (RATIFY trial)
*FLT3*-ITD or TKD AML	*FLT3* kinase	Gilteritinib	Relapsed/refractory	FDA-approved	Median OS 9.3 vs. 5.6 months (ADMIRAL)
*FLT3*-ITD AML	*FLT3* kinase (type II)	Quizartinib	Frontline with 7 + 3	FDA-approved	OS 31.9 vs. 15.1 months (QuANTUM-First)
*FLT3*-WT AML (*FLT3*-like signature)	*FLT3* signaling	Quizartinib	Frontline (investigational)	Phase III	Improved EFS and OS in *FLT3*-like subset (QUIWI)
*IDH1*-mutated AML	*IDH1*, inhibition of 2HG generation	Ivosidenib	Relapsed/refractory	FDA-approved	CR or CRh 30.4%, ORR 41.6%, median duration of response 8.2 months
*IDH2*-mutated AML	*IDH2*, inhibition of 2HG generation	Enasidenib	Relapsed/refractory	FDA-approved	ORR 40.3%, median duration of response 5.8 months
*TP53*-mutated AML	p53 reactivation	Eprenetapopt (APR-246)	HMA combinations	Phase III failed	Higher response without OS benefit
*TP53*-mutated AML	CD47 innate immune checkpoint	Magrolimab	HMA combinations	Phase III failed	CR 57% but no OS benefit, Increasedtoxicity
*TP53*-mutated AML	p53 Y220C pocket	PC14586 (Rezatapopt)	Early phase	Phase I	Selective activity in Y220C *TP53* AML

## Data Availability

No new data were created or analyzed in this study.
